# *Notes from the Field*: Preliminary Results After Implementation of a Universal Treatment Program (Test and Start) for Persons Living with HIV Infection — Lesotho, October 2015–February 2017

**DOI:** 10.15585/mmwr.mm6630a4

**Published:** 2017-08-04

**Authors:** Amee M. Schwitters

**Affiliations:** 1 Division of Global HIV and TB, Center for Global Health, CDC.

Lesotho, a small, mountainous country completely surrounded by the Republic of South Africa, has a population of approximately 2 million persons with an estimated gross national income of $1,280 per capita; 73% of the population resides in rural areas (*1*). Lesotho has a generalized human immunodeficiency virus (HIV) epidemic (*2*). In 2014, the prevalence of HIV infection among persons aged 15–49 years was 24.6%, with an incidence of 1.9 new infections per 100 person-years of exposure (*3*). As the leading cause of premature death, HIV/AIDS (acquired immunodeficiency syndrome) has contributed to Lesotho’s reporting the shortest life expectancy at birth among 195 countries and territories (*4*). In 2015, antiretroviral therapy (ART) coverage among HIV-positive persons in Lesotho was estimated to be 42% (*5*). In April 2016, Lesotho became the first country in sub-Saharan Africa to adopt the World Health Organization (WHO) recommendations for universal initiation of antiretroviral therapy for all HIV-positive persons, regardless of CD4 count (known as the “Test and Start” program or approach), with nationwide implementation occurring in June 2016 (*6*,*7*). Before implementation of Test and Start, many persons living with HIV infection in Lesotho were not eligible to initiate treatment until their CD4 count was <500 cells/mm^3^.

The President’s Emergency Plan for AIDS Relief (PEPFAR) supports treatment activities in 120 sites (114 public and six private) in five of Lesotho’s 10 districts. The five districts supported by PEPFAR are home to approximately 75% of all HIV-positive persons in the country. Sites that have a minimum of 200 persons undergoing treatment for HIV infection are eligible for inclusion in the program.

In the 8 months preceding implementation of Test and Start (October 2015–May 2016), 14,948 HIV-positive persons were initiated on ART at the 120 PEPFAR-supported sites. In the 9 months since implementation of Test and Start (June 2016–February 2017), 30,146 persons were initiated on ART at the same sites, representing a 79% increase in the average monthly number of HIV-positive persons who were initiated on treatment (Figure). During the same time, treatment coverage increased 80% among males and 79% among females. The average monthly increases in coverage among persons aged <15 years, 15–24 years, and ≥25 years were 72%, 84%, and 79%, respectively. The average monthly increase in coverage varied by PEPFAR-supported district, ranging from 62% in Mohale’s Hoek to 109% in Leribe. In fiscal year 2018 an additional 32 sites that have ≥200 HIV-infected persons undergoing treatment will be supported by PEPFAR in the five districts. Information is not currently available on the percentage of HIV-positive persons newly initiated on treatment who were previously known to be infected, but who did not meet the eligibility criteria for treatment initiation, and the percentage of persons in whom HIV infection was newly diagnosed.

**FIGURE:**
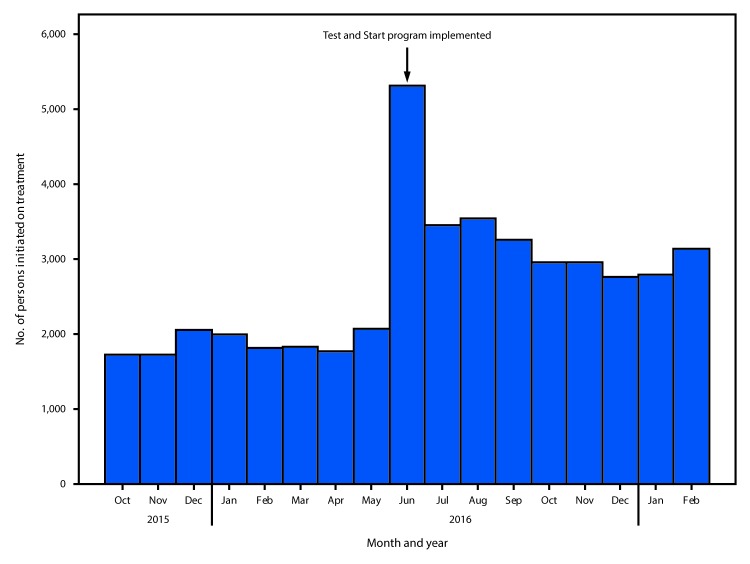
Number of persons infected with human immunodeficiency virus (HIV) initiated on antiretroviral treatment before and after implementation of the universal HIV treatment program Test and Start, by month — five PEPFAR-supported districts, Lesotho, October 2015–February 2017. **Abbreviation:** PEPFAR = President’s Emergency Plan for AIDS Relief.

Aligned with the Joint United Nations Programme on HIV and AIDS strategy,* PEPFAR’s goal in Lesotho is 80% ART coverage among HIV-positive persons in five districts to achieve epidemic control (i.e., the point at which newly diagnosed HIV infections have decreased and fall below the number of AIDS-related deaths) (*8*). The partnership between the Lesotho Ministry of Health, PEPFAR, and implementing partners has resulted in promising preliminary results after implementation of Test and Start; sustained progress will represent a critical step toward achieving epidemic control. Successful implementation of Test and Start in all sites and districts across Lesotho, coupled with additional measures to retain HIV-positive persons newly initiated on treatment, could help maximize the success of Test and Start and the benefit of treatment to prevent new HIV cases.

## Footnotes

* The Joint United Nations Programme on HIV and AIDS strategy is the following: 90% of all persons living with HIV will know their HIV status, 90% of all persons with diagnosed HIV infection will receive sustained antiretroviral therapy, and 90% of all persons receiving antiretroviral therapy will achieve viral suppression.
